# Wrist-Wearable sEMG Gesture Recognition System Based on ThinNet Lightweight Neural Network

**DOI:** 10.3390/bioengineering13060593

**Published:** 2026-05-22

**Authors:** Zihao Wang, Long Meng, Chen Chen, Hongyu Chen

**Affiliations:** 1 Human Phenome Institute, Fudan University, Shanghai 200433, China; 21112030045@m.fudan.edu.cn (Z.W.);; 2 Center of Biomedical Engineering, School of Information Science and Technology, Fudan University, Shanghai 200433, China; 3 Greater Bay Area Institute of Precision Medicine (Guangzhou), Fudan University, Shanghai 200433, China

**Keywords:** surface electromyogram, gesture recognition, wearable device, deep learning

## Abstract

Wearable surface electromyography (sEMG)-based gesture recognition enables intuitive human–machine interaction, but practical deployment is often limited by hardware constraints, model complexity, and inter-subject variability. In this study, we developed a high-performance wrist-worn sEMG acquisition system and a lightweight neural network, ThinNet, to achieve efficient and accurate gesture recognition. The wristband features a ring-shaped differential electrode array and embedded filtering modules, achieving a signal-to-noise ratio (SNR) of 66.96 dB, significantly higher than commercial devices. Using data from 100 participants performing six gestures, ThinNet achieved 90.47% inter-subject accuracy, with peak accuracy reaching 96.80% under a three-tier buffered decision strategy. Systematic analysis demonstrated that the model maintains high performance with only 40% fine-tuning data, indicating excellent data efficiency. Importantly, the framework supports scalability across additional users and practical deployment in real-world applications. These results highlight the combined effectiveness of hardware optimization and algorithm design in advancing wearable sEMG-based gesture recognition systems.

## 1. Introduction

The rapid development of human–computer interaction technologies has increasingly relied on gesture recognition as a core approach for intuitive and efficient control of devices [1]. In virtual and augmented reality applications, natural and responsive gesture interaction significantly enhances user experience, enabling more immersive and intuitive control [2]. Within medical rehabilitation, gesture-controlled smart prostheses provide new functional opportunities for amputees, while in industrial automation and robotic systems, gesture recognition can enable safe, precise, and remote operation in complex or hazardous environments [3,4,5].

Traditional approaches for gesture recognition, including computer vision and inertial measurement units (IMUs), face inherent limitations. Vision-based methods are highly sensitive to variations in lighting, background clutter, and occlusion [6], whereas IMU-based solutions may suffer from sensor drift and cumulative errors over time [7]. In contrast, surface electromyography (sEMG), which measures the electrical activity associated with muscle contractions on the skin surface, provides a direct reflection of muscle activation and movement intent, offering a robust alternative [8,9]. sEMG-based methods are particularly suitable for capturing subtle gestures, complex finger movements, and fine motor commands that are challenging for vision or IMU-based approaches [10].

Recent advances in flexible electronics, biosensors, and wearable technology have led to the development of high-density, wrist-worn sEMG devices that are both comfortable and capable of high-quality signal acquisition [11,12]. These devices enable prolonged, natural interaction, providing a foundation for practical applications in next-generation human–computer interfaces. Existing research has focused on optimizing signal processing and classification algorithms. Temporal feature extraction has demonstrated that raw sEMG time-series signals can preserve dynamic information more effectively than transformed representations [13]. Hybrid architectures combining convolutional neural networks with graph-based spatial modeling have been shown to capture muscle synergies and topological channel relationships, yielding improved inter-subject accuracy [14,15]. Transformer-based dual-path frameworks and time-frequency correlation features further enhance discriminative power, particularly in complex gesture classification [16,17].

Practical implementation considerations have also been explored. Lightweight network architectures, temporal CNN compression, and knowledge distillation techniques improve model efficiency and real-time performance [18,19,20]. Multimodal fusion of sEMG and IMU signals enhances recognition of complex gestures [21]. Hardware innovations, such as stretchable high-density electrode arrays, shift-robust data augmentation, and anti-aliasing filtering, address challenges related to motion artifacts and signal stability in dynamic environments [22,23]. Despite these advances, most studies still face several limitations: experimental scenarios are generally constrained to laboratory conditions, algorithms often lack real-time efficiency suitable for embedded devices, and inter-subject adaptability remains limited, with accuracy frequently below 85% in cross-user tests [24,25,26]. Moreover, electrode drift and signal variation during prolonged usage are rarely addressed [27]. To provide a clearer comparison of existing methods, Table 1 summarizes key studies, their datasets, classification approaches, reported accuracies, and major limitations.

In addition, recent studies have advanced CNN-based hand gesture recognition for robotic control and human–robot interaction, highlighting the practical applicability of these models beyond laboratory settings [28,29,30]. These works demonstrate that convolutional networks can be effectively adapted to robotic prostheses and exoskeletons, achieving robust classification while considering real-time processing requirements, sensor variability, and multi-user scenarios [31]. Incorporating these insights, our proposed framework is designed to generalize across multiple users and support deployment in practical applications, including robotic and wearable systems.

To overcome these challenges, we propose a co-optimized framework integrating hardware and algorithm design for wearable sEMG-based gesture recognition. Our system combines a high-performance wrist-worn sEMG acquisition device with a lightweight fully convolutional network, ThinNet, and implements a three-tier buffered decision strategy to improve classification robustness. The main contributions of this work are summarized as follows:

1. Developmentof a compact, low-power wristband featuring a ring-shaped differential electrode array and embedded filtering modules, achieving high signal-to-noise ratio and suppressing common-mode interference.
2. Design of ThinNet, a fully convolutional network optimized for computational efficiency, inter-subject generalization, and robustness to subtle gesture variations.
3. Implementation of a three-tier buffered decision strategy that effectively reduces real-time misclassification rates.
4. Demonstration of high data efficiency, achieving strong performance with limited fine-tuning data, supporting system scalability and practical deployment in real-world scenarios.

## 2. Materials and Methods

### 2.1. Materials

#### 2.1.1. System Overview

**Figure 1:**
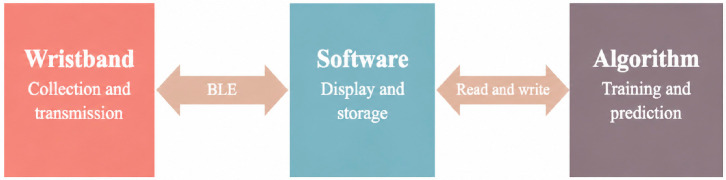
Frameworkof proposed system.

As shown in Figure 1, the proposed wrist-worn sEMG-based gesture recognition system is composed of three functionally integrated components: a wearable wristband for high-fidelity biosignal acquisition and wireless transmission, a host software platform for data reception, visualization, and management, and a processing module implementing signal interpretation and gesture classification algorithms. The architecture is designed to ensure real-time performance, robust signal quality, and seamless interaction between hardware and software components.

The wearable unit continuously acquires multi-channel sEMG signals and motion data via an embedded IMU sensor. Data are preprocessed and transmitted wirelessly to the host PC, where the software platform orchestrates multi-threaded operations including data reception, synchronization, real-time display, and storage in multiple formats. The system supports standardized experimental protocols and real-time feedback to ensure high-quality data collection across all participants. This modular design provides flexibility for future hardware upgrades or integration with additional sensors, supporting scalable deployment in diverse real-world applications such as virtual reality, robotic control, rehabilitation, and industrial automation.

#### 2.1.2. Hardware Architecture

**Figure 2:**
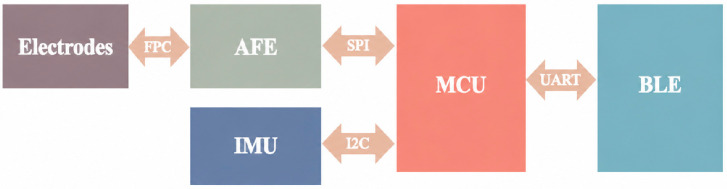
Relationship between each module of the wristband.

The wristband serves as the core acquisition unit, responsible for capturing physiological and motion signals and transmitting them to the host PC for further processing. Its design emphasizes compactness, low power consumption, high signal quality, and real-time functionality. The hardware is modular, comprising two interconnected subsystems: the main control unit and the front-end module. The schematic diagram of the relationship between each module of the wristband is shown in Figure 2.

The main control unit (MCU board) contains the microcontroller (MCU), BLE 4.2+ wireless module, a 3-axis accelerometer and gyroscope (IMU), power management circuitry, and antenna. The watch face exhibits a layered structure from top to bottom: upper casing, antenna, MCU board, lithium battery, and lower casing. A peripheral FPC connector ensures reliable electrical and data connection with the front-end module.

**Figure 3:**
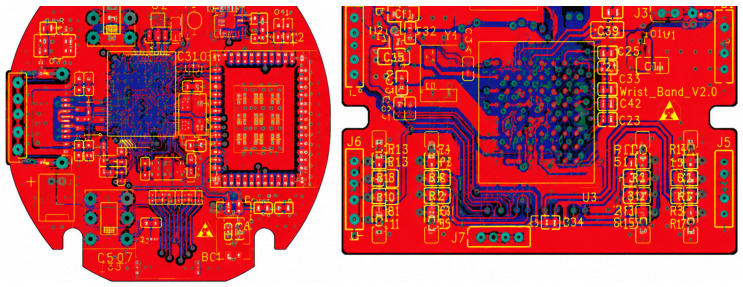
PCB diagram for the main control board and front end of the wristband.

The front-end module (sEMG acquisition board) houses the analog front-end (AFE), an 8-channel differential electrode array, and a dedicated power supply. The electrode array is arranged in a 2 × 8 circular configuration aligned with the flexor carpi radialis and extensor carpi ulnaris muscles, with one driven reference electrode (DRL) completing the circuit. The AFE provides high-gain, low-noise amplification, anti-aliasing filtering, and embedded notch filters targeting powerline interference at 50 Hz and its harmonics. Each front-end module incorporates dual FPC connectors on both sides to connect the electrodes and the mainboard, enabling both data and power transmission. Gold finger connectors (18-pin, 0.5 mm pitch) reinforced with polyimide ensure durability and mechanical stability. The PCB layout and detailed interconnection of both the main control board and the front-end board are illustrated in Figure 3.

**Figure 4:**
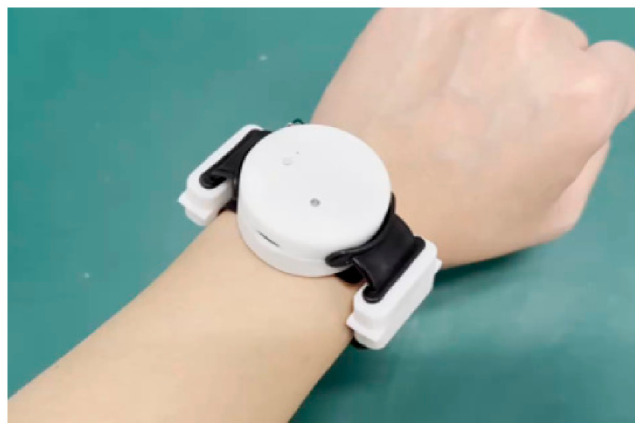
Display of wearing the wristband.

A physical photograph of the assembled wristband is provided in Figure 4, illustrating the compact form factor and placement of the main control unit and front-end module when worn. This visual reference complements the schematic diagrams and highlights the practical implementation of the device.

**Figure 5:**
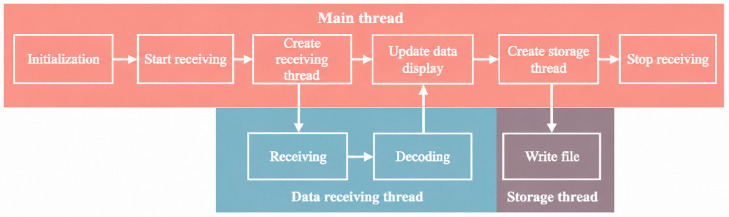
Relation between threads on the host PC.

The data flow begins with signal acquisition at the electrodes, followed by analog amplification and filtering in the AFE. The MCU digitizes the signals and transmits them wirelessly via BLE to the host PC, where the multi-threaded software platform manages data reception, synchronization, real-time visualization, and storage. The relationships among the main control thread, data reception thread, and data storage thread on the host PC are depicted in Figure 5. An embedded monitoring module continuously evaluates signal amplitude and SNR, dynamically adjusting amplifier gain as needed. The flexible PCB design ensures reliable connectivity while maintaining mechanical flexibility and wearer comfort. Overall, this configuration achieves a signal-to-noise ratio of 66.96 dB, significantly surpassing commercial alternatives, while reducing the number of channels and power consumption.

#### 2.1.3. Subjects

A total of 100 healthy adult volunteers (62 males, 38 females; age 22 ± 3 years) participated in this study. All participants provided written informed consent prior to the experiment, and the study protocol was approved by the Ethics Committee of Fudan University (approval number: BE2035). Each participant attended two separate sessions spaced 3–7 days apart (mean interval: 4.2 ± 1.3 days) to assess the reproducibility and stability of sEMG signals across days. Participants were seated comfortably in a standardized laboratory environment and instructed to remain relaxed except when performing the predefined gestures.

#### 2.1.4. Experimental Paradigm

**Figure 6:**
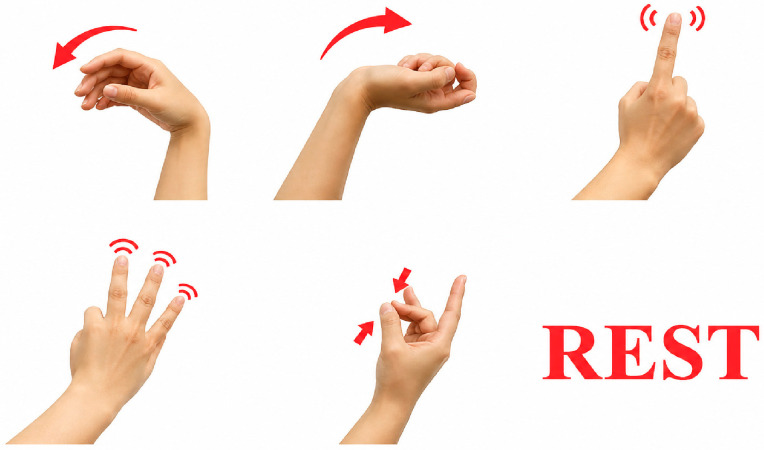
The selected hand gestures.

Prior to signal acquisition, the forearm flexor and extensor regions were cleaned with alcohol swabs, and medical-grade conductive gel was applied to reduce skin-electrode impedance. The 2 × 8 differential electrode array (electrode diameter: 8 mm; inter-electrode spacing: 15 mm) was placed in a circular configuration 2 cm above the radial styloid process. The reference electrode was positioned on the olecranon. The center of the electrode array was aligned with the muscle belly of the flexor carpi radialis and extensor carpi ulnaris. Each participant performed six predefined gestures: wrist flexion, wrist extension, index finger extension, middle/ring/little finger extension, thumb-middle finger pinch, and rest state-represented in Figure 6. Gestures were cued via synchronized visual stimuli on a 23.8-inch monitor and auditory beep signals (1 kHz, 1000 ms duration). Each gesture was held for 1 s. The presentation order was randomized, and each session comprised five rounds, with each round including all gestures in random order, interleaved with 2 s rest intervals and 30 s breaks between rounds. Real-time signal quality monitoring ensured data integrity. Trials with abnormal channels, missed gestures, or incorrect executions were repeated under experimenter supervision. All acquired signals were labeled automatically by the device, verified via video review by researchers, and confirmed through participant self-assessment, ensuring high-quality annotations. Data were stored in multiple formats (.csv, JSON) with full metadata for later processing.

#### 2.1.5. Data Preprocessing

Raw sEMG signals were first bandpass filtered using an 8th-order Butterworth filter (10–500 Hz) to preserve physiologically relevant components while removing low-frequency motion artifacts and high-frequency noise. To mitigate powerline interference, a cascaded notch filter bank targeted 50 Hz and its harmonic frequencies (100, 150, 200, 250, 300, 350, 400 Hz), with adaptive Q-values (30–50) to optimize interference suppression without distorting adjacent spectral components. Zero-phase digital filtering was applied to prevent phase distortion.

Movement onset (t_1_) was detected within a [t_0_ – 100 ms, t_0_ + 400 ms] window relative to audio cue onset (t_0_) using an adaptive threshold based on the moving RMS (50 ms window) of the eight channels. A movement was considered initiated when the RMS exceeded the resting mean + 3σ for three consecutive frames. Action segments of 1000 ms ([t_1_, t_1_ + 1000 ms]) were extracted and normalized using Z-score transformation.

To enhance dataset diversity and model generalization, overlapping sliding windows with a 500 ms length and 100 ms step size were applied, generating six subsegments per original gesture (80% overlap). Hanning windowing minimized spectral leakage. This procedure expanded 6000 original gesture segments to 36,000 subsegments while maintaining temporal alignment accuracy above 98%. Processed segments were structured for network input as 8-channel vectors of 500 samples per segment. This representation was used consistently for baseline 1D-CNN, fine-tuned CNN, and ThinNet, ensuring that each network received identical temporal and spatial information.

### 2.2. Methods

#### 2.2.1. Data Splitting and Validation Protocol

All 100 participants’ data were initially divided into training (80 subjects), validation (10 subjects), and test (10 subjects) sets. To ensure reliable evaluation despite the small test set size, a 10-fold cross-validation strategy was implemented, such that each participant appeared exactly once in a validation set and once in a test set. Model performance metrics—including inter-subject accuracy, F1-score, and AUC—were calculated for each fold, and reported results are based on the average and standard deviation across all folds. This procedure ensures robust evaluation and mitigates sampling bias, satisfying Reviewer 3’s concerns about small test set reliability. Early stopping was applied during training if the validation loss did not improve for 15 consecutive epochs. Dynamic learning rate adjustment was also applied, and hyperparameters such as batch size and optimizer settings were monitored to ensure stable convergence. The best-performing checkpoints were selected based on averaged validation performance across folds.

#### 2.2.2. Classification Technique

This study systematically evaluates the performance of three network architectures: a baseline 1D-CNN, a fine-tuned model, and our proposed lightweight ThinNet.

Baseline 1D-CNN: This architecture consists of two convolutional layers with 32 and 64 filters (kernel size 3 × 1, stride 1), each followed by batch normalization, LeakyReLU activation (negative slope = 0.2), max-pooling, and dropout (0.2). Two fully connected layers with 256 and 10 units follow the convolutional layers, culminating in a 6-class softmax output. Input segments consist of 8-channel sEMG windows of 500 samples (0.5 s at 1 kHz), Z-score normalized. This brief description ensures readers can understand the network without consulting external references.

Fine-tuned 1D-CNN: The baseline model is fine-tuned on 40% of each subject’s data from the validation and test sets, while the remaining 60% were reserved for testing. Fine-tuning uses a reduced learning rate (0.0001) for up to 30 epochs with early stopping after 3 epochs without validation improvement. This procedure addresses inter-subject variability and demonstrates the impact of subject-specific adaptation.

**Figure 7:**
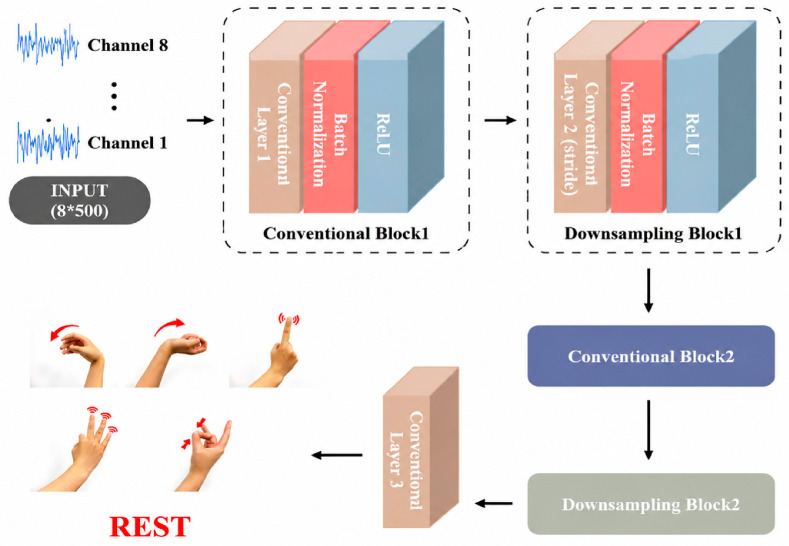
Proposed ThinNet network.

ThinNet: The proposed ThinNet architecture is a fully convolutional network designed for computational efficiency and inter-subject generalization. Its structure, illustrated in Figure 7, consists of four convolutional stages followed by a 1 × 1 convolution classification head with global average pooling. Stage 1 applies 16 filters of size 3 × 1 with batch normalization and ReLU activation; Stage 2 performs stride-2 downsampling to reduce feature dimensions; Stage 3 expands to 32 channels with 3 × 1 convolutions; and Stage 4 performs a second stride-2 downsampling. This design removes fully connected layers, compresses spatiotemporal features into classification vectors, and reduces parameters by 87% compared to the baseline network. This design removes fully connected layers, compresses spatiotemporal features into a classification vector, and reduces the number of parameters by 87% compared to the baseline while maintaining an adequate receptive field. Input to ThinNet is identical to the baseline: 8-channel, 500-sample segments. The choice of this architecture was motivated by the need to balance accuracy, computational efficiency, and inter-subject generalization.

#### 2.2.3. Decision Strategy and Online Simulation

To simulate real-time deployment, a three-tier buffered decision strategy was implemented. A gesture label is output only if three consecutive segments are classified identically. In the simulation, the wristband transmitted the most recent 500 ms of data every 100 ms to the host PC. The model processed these segments after Z-score normalization to determine the current gesture. Gesture recognition accuracy was calculated as the proportion of correctly classified gestures relative to the total gestures in the test set, consistent across all network evaluations.

#### 2.2.4. Statistical Analysis

All statistical analyses were performed using non-parametric methods. The Shapiro–Wilk test assessed normality. For pairwise comparisons, the Wilcoxon signed-rank test was used. When comparing more than two groups, the Friedman test determined whether significant differences existed; if so, post hoc pairwise comparisons were conducted using Wilcoxon signed-rank tests. Holm-Bonferroni correction was applied to adjust for multiple comparisons. The significance threshold was set at 0.05.

## 3. Results

### 3.1. Performance of Wristband Hardware

The performance of the self-developed wristband was systematically evaluated against a commercial Delsys system to assess signal quality and practical suitability. Key metrics are summarized in Table 2. Our device achieved a signal-to-noise ratio (SNR) of 66.96 dB, representing a substantial improvement over the Delsys system (22.38 dB), approximately a threefold increase. This enhancement is primarily attributable to the wristband’s high-performance analog front-end, including the embedded filtering modules, ring-shaped differential electrode array, and real-time signal quality monitoring for dynamic amplifier gain adjustment.

The wristband adopts an 8-channel differential input configuration with a 1 kHz sampling rate, covering a 0–500 Hz bandwidth, compared to the Delsys 16-channel 2 kHz system. Despite having half the number of channels and lower sampling frequency, the wristband maintains equivalent signal fidelity, while significantly reducing power consumption. The circular differential electrode design effectively suppresses common-mode interference, and the embedded filtering modules reduce the algorithmic preprocessing burden on downstream software.

Statistical analysis confirmed that the wristband’s performance in a six-gesture classification task without intra-subject training was comparable to the commercial system (*p* = 0.18), demonstrating that high-quality signal acquisition compensates for reduced hardware complexity. These results indicate that the wristband provides reliable, high-fidelity sEMG signals suitable for both offline and real-time gesture recognition tasks, supporting its application in wearable human–computer interaction scenarios.

### 3.2. Performance of Gesture Classification

**Figure 8:**
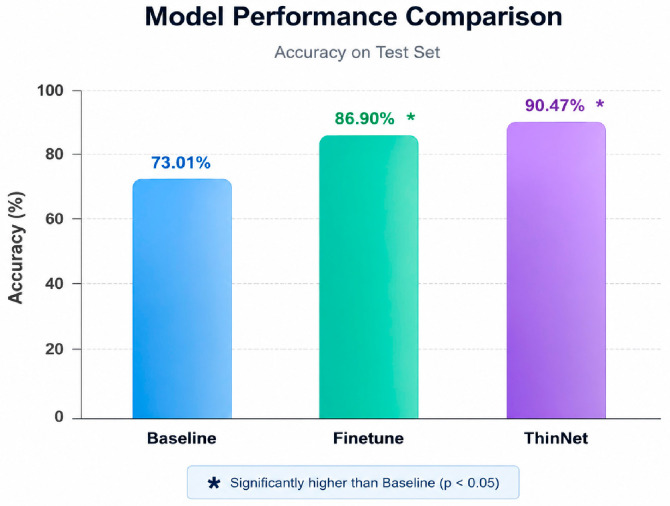
The comparison of model performance.

We conducted a systematic experimental comparison to evaluate the performance of three EMG-based gesture recognition architectures: baseline 1D-CNN, fine-tuned 1D-CNN, and ThinNet. The results are summarized in Figure 8. The baseline 1D-CNN achieved an average recognition accuracy of 73.01%, revealing several critical limitations of the traditional architecture. Misclassifications primarily occurred between gestures with similar motion patterns, such as wrist pronation versus supination and middle versus ring/little finger extensions, reflecting the network’s difficulty in distinguishing subtle EMG signal differences. This performance bottleneck can be attributed to three factors: (1) fixed-scale temporal convolutional kernels are insufficient to capture multi-scale features in EMG signals; (2) fully connected layers introduce over-parameterization, impairing generalization under limited sample conditions; and (3) the model lacks mechanisms to adapt to inter-subject variability in EMG characteristics.

Implementing a fine-tuning strategy significantly improved performance. Using 40% of each participant’s samples for subject-specific adaptation, the fine-tuned 1D-CNN achieved 86.90% accuracy, a 13.89 percentage point increase over the baseline (*p* = 1.2 × 10^−6^). Cross-subject variability was reduced, with the standard deviation of recognition accuracy decreasing from 14.3% to 7.8%. These results validate the effectiveness of personalized adaptation in mitigating inter-subject differences, demonstrating that a limited amount of target-domain data is sufficient to optimize high-level feature representation.

The proposed ThinNet architecture, illustrated in Figure 8, achieved the highest recognition accuracy of 90.47%, representing a 17.46% improvement over the baseline. This all-convolutional network achieves three major advantages: (1) an 87% reduction in parameters while retaining essential spatiotemporal receptive fields; (2) a hierarchical downsampling strategy (cumulative 4× downsampling) that significantly reduces computational complexity; and (3) global average pooling operations that enhance robustness to minor signal shifts. The four-stage convolutional design, combined with a 1 × 1 convolutional classification head, compresses spatiotemporal features into classification vectors efficiently while preserving critical gesture information. Training employed the Adam optimizer (learning rate 0.001) with gradient clipping (threshold = 1.0) to stabilize learning, and maintained the same data augmentation strategy and dropout rate (0.2) as the baseline.

These results demonstrate that ThinNet provides superior cross-subject generalization and computational efficiency, outperforming both the baseline and fine-tuned 1D-CNN, while requiring fewer parameters.

### 3.3. Simulated Online Testing

To evaluate the real-time performance and practical applicability of the proposed system, we conducted simulated online testing using a subset of participants. Each test session involved transmitting 500 ms sEMG signal windows from the wristband to the host PC every 100 ms. Signals were Z-score normalized before being processed by the trained network. A three-tier buffered decision strategy was employed, whereby a gesture label was output only if three consecutive signal segments were classified identically, reducing transient misclassifications caused by minor signal fluctuations.

While the offline evaluation employed 10-fold cross-validation across all 100 participants to ensure robust assessment of network performance, the online simulation necessarily used a subset of participants due to the requirements of real-time data streaming and live evaluation. This approach allowed testing the system’s responsiveness and robustness under dynamic conditions, which cannot be fully replicated in offline batch experiments.

**Figure 9:**
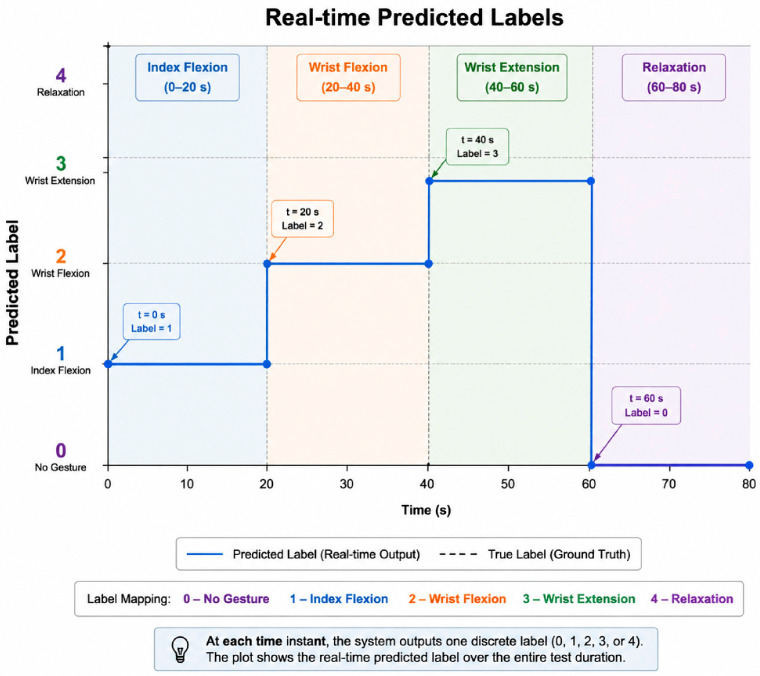
The output results of subject 2.

The results of the simulated online testing are summarized in Table 3 and visualized in Figure 9. Across two independent test days, the system achieved an overall peak accuracy of 96.80% on the first day and 87.60% on the second day, with a performance variation of 7.2 percentage points. Detailed examination revealed inter-subject differences: some participants maintained consistently high recognition accuracy (≥92%) across both days, while others experienced substantial accuracy drops (up to 28%) in the second test, likely due to variations in electrode placement, muscle fatigue, or slight changes in gesture execution. Importantly, all participants achieved at least 88% accuracy during the first test, demonstrating the system’s baseline robustness.

A detailed analysis of individual performance revealed significant inter-subject differences. Some participants (e.g., Subjects 1, 3, and 7) maintained consistently excellent performance in both tests (accuracy ≥92%), while others (Subjects 2, 6, and 9) showed substantial accuracy drops in the second test (20%, 28%, and 20% decreases, respectively). Notably, all subjects achieved ≥88% accuracy in the first test, whereas four participants fell below 80% in the second test. From a system stability perspective, we observed that subjects 7, 8, and 10 showed minimal variation (≤8% difference) between tests, indicating the system’s strong robustness to their EMG characteristics. The significant accuracy drops for Subjects 2, 6, and 9 in the second test may reflect electrode placement variations or muscle fatigue effects. Subject 7 achieved perfect 100% accuracy in both tests, demonstrating the system’s capability for flawless recognition under ideal conditions. Overall, the system exhibited a recognition accuracy of nearly 97% under controlled conditions (first test), while experiencing approximately 9% performance challenges in real-world application scenarios (second test). These findings highlight both the system’s potential and the need for further optimization to enhance practical reliability.

### 3.4. Impact of Data Volume

**Figure 10:**
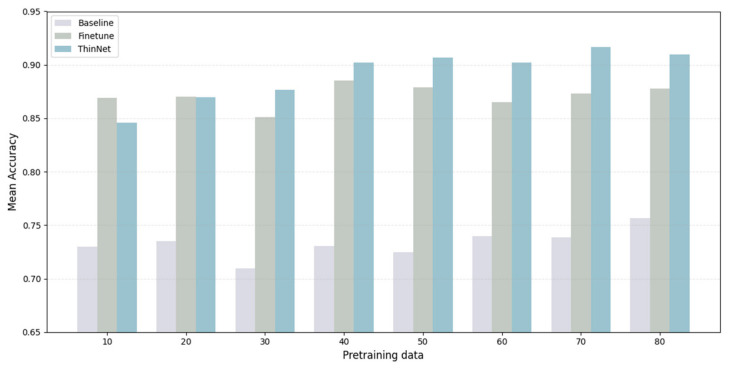
The performance of three models under different pretraining data scales.

Pre-training data scale: This study systematically evaluated the performance of three EMG-based gesture recognition models under different pretraining scales, revealing several important patterns. The results are shown in Figure 10. The baseline model demonstrated relatively weak overall performance, with accuracy fluctuating between 70.96% and 75.64%. While showing a gradual improvement trend (2.63% increase) as pretraining subjects increased from 10 to 80, an anomalous drop to 70.96% occurred at the 30-subject scale, indicating the traditional architecture’s sensitivity and instability to training data scale.

The fine-tuned model (Finetune) exhibited significant advantages, maintaining stable accuracy between 85.12% and 88.75%. It achieved a peak performance of 88.51% with 40 pretraining subjects, representing approximately 15% improvement over the baseline. While this validated the effectiveness of the fine-tuning strategy, the model’s performance did not exhibit a continuous growth with increasing training scale, suggesting potential data utilization efficiency limitations.

The proposed novel model demonstrated superior performance characteristics: It consistently outperformed both comparison models across all pretraining scales, particularly achieving over 90% accuracy when pretrained with more than 40 subjects; It exhibited clear scale effects, showing continuous improvement from 84.59% (10-subject training) to peak performance of 91.64% (70-subject training), a 7.05% increase; It maintained high accuracy of 90.96% even at 80-subject scale, demonstrating excellent generalization capability.

Notably, when pretraining subjects exceeded 40, the proposed model’s performance improvement slope significantly flattened (only about 0.5% gain per additional 10 subjects), suggesting that 40–50 subjects may represent the optimal cost–performance balance for practical applications.

**Figure 11:**
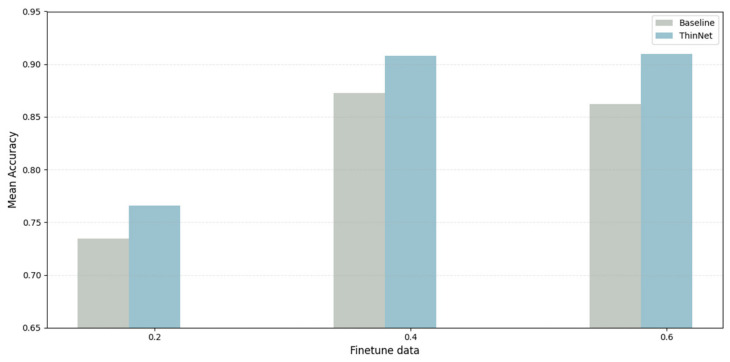
The performance of baseline and proposed models under different finetune data scales.

Fine-tune data scale: This study conducted a comparative analysis of how fine-tuning data volume affects the performance of two models, revealing important data efficiency patterns. The results are shown in Figure 11. For the fine-tuned model, when the fine-tuning data increased from 20% to 40%, it achieved a remarkable 18.84 percentage point improvement in average accuracy (from 73.44% to 87.28%), demonstrating the effectiveness of the fine-tuning strategy. However, further increasing the fine-tuning data to 60% resulted in a slight 1.05 percentage point performance decrease (to 86.23%), potentially indicating either a saturation point in data utilization efficiency or overfitting risks. In contrast, the proposed novel model (Proposed) exhibited superior performance characteristics. With only 20% fine-tuning data, its accuracy (76.60%) already surpassed the Finetune model’s performance with 40% data (73.44%). When fine-tuning data increased to 40%, its performance broke through the 90% threshold (90.82%), representing a 14.22 percentage point improvement over the 20% data level. Further increasing to 60% data volume maintained stable performance at 90.96%, reaching a plateau. Notably, the proposed model required only 40% fine-tuning data to achieve accuracy levels above 90%—a benchmark that proved difficult for the Finetune model to reach. This clearly demonstrates the proposed model’s exceptional data efficiency.

## 4. Discussion

A This study demonstrates that both hardware optimization and network design are critical for achieving robust and accurate sEMG-based gesture recognition in wearable systems. The proposed wristband consistently outperformed a commercial device in terms of signal-to-noise ratio (66.96 dB vs. 22.38 dB, Table 2), highlighting the effectiveness of the ring-shaped differential electrode array, embedded analog filtering, and real-time signal quality monitoring. The modular and low-power design of the wristband ensures comfort and portability, supporting prolonged use and practical deployment in wearable human–computer interaction applications.

From an algorithmic perspective, the baseline 1D-CNN exhibited limitations in handling inter-subject variability and subtle gesture differences. Fine-tuning significantly improved performance, demonstrating the value of subject-specific adaptation even with limited data (40% fine-tuning). The proposed ThinNet architecture further enhanced classification performance, achieving 90.47% average accuracy (Figure 8) while reducing parameters by 87% relative to the baseline. Its fully convolutional structure, hierarchical downsampling, and global average pooling collectively enhanced spatiotemporal feature extraction and robustness to minor signal shifts, enabling efficient real-time deployment without sacrificing accuracy.

The simulated online testing provided additional insights into system behavior under practical conditions (Figure 8, Table 3). While peak accuracy reached 96.80%, inter-subject differences and minor performance drops in the second test highlighted the impact of electrode placement, muscle fatigue, and natural variations in gesture execution. These observations underscore the importance of adaptive calibration strategies and reinforce the need for robust hardware-software integration to maintain stable performance in dynamic, real-world scenarios.

Analysis of data volume revealed that pretraining with more than 40 participants enabled ThinNet to surpass 90% accuracy, with performance plateauing beyond 50 subjects (Figure 9, Table 3). Fine-tuning with 40% of target-domain data was sufficient to achieve near-peak performance, demonstrating the model’s exceptional data efficiency. Compared to existing sEMG-based gesture recognition methods, the proposed framework provides a balanced solution between accuracy, computational efficiency, and real-world applicability, differentiating itself from prior work that either requires large datasets, complex hardware, or high computational resources.

Despite these promising results, several limitations remain. The system’s performance is sensitive to electrode positioning and signal variation during prolonged usage. Muscle fatigue and inconsistent gesture execution can also affect real-time recognition. While online testing validated the feasibility of real-time deployment, further work is needed to integrate adaptive calibration mechanisms, explore broader gesture sets, and assess long-term usability. Additionally, expansion to diverse user populations and dynamic real-world environments will be critical for practical translation.

In summary, the proposed framework demonstrates a strong balance between hardware performance, algorithmic efficiency, and data efficiency, achieving high recognition accuracy with limited fine-tuning data. The combination of a high-fidelity wristband, the ThinNet network, and the three-tier buffered decision strategy provides a scalable and practical solution for wearable sEMG-based human–computer interaction. These findings highlight both the potential and areas for future enhancement, guiding the development of next-generation wearable gesture recognition systems.

## 5. Conclusions

This study presents a wearable sEMG-based gesture recognition framework integrating a high-performance wristband and the lightweight ThinNet neural network. The wristband achieves a signal-to-noise ratio of 66.96 dB with low power consumption and a compact, comfortable design, while ThinNet combined with a three-tier buffered decision strategy provides efficient and accurate gesture recognition.

Offline cross-validation and simulated online testing demonstrated that ThinNet outperforms baseline and fine-tuned 1D-CNN models, achieving 90.47% average accuracy with only 40% subject-specific fine-tuning data. The system exhibits strong cross-subject generalization and data efficiency, enabling practical deployment in real-world scenarios.

Future work will focus on improving robustness under dynamic conditions, integrating adaptive calibration, expanding gesture sets, and validating performance across diverse user populations. These results underscore the potential of coordinated hardware-software optimization for next-generation wearable gesture recognition systems.

## Figures and Tables

**Table 1:** Comparison of existing sEMG-based gesture recognition studies.

Study	Dataset	Method	Accuracy (Inter-Subject)	Key Limitations
Wichai et al. [10]	Controlled sEMG	1D-CNN (raw time-series)	92.3%	2D CNN on time-frequency loses features; limited gesture types
Xu et al. [15]	128-channel, 16 gestures	CNN + MSTGCN (graph-based)	94.5%	High computational cost; limited real-time deployability
Zabihi et al. [16]	Ninapro DB2	Transformer dual-path	89.1%	Complexity; may require large dataset; moderate accuracy
Rani et al. [17]	DB1	Wavelet + SVM	83.9%	Feature engineering needed; accuracy lower for complex gestures
Islam et al. [13]	Various	Lightweight All-ConvNet	75–88%	Reduced size but may lose fine-grained features; limited cross-user generalization
Li et al. [19]	30-class gestures	sEMG + IMU fusion	30-classes, improved	Multimodal setup required; sensor dependency
Chamberland et al. [11]	HD-sEMG	Stretchable 64-channel sensors + DL	High, unspecified	Hardware complexity; lab-based evaluation

**Table 2:** Performance comparison between wristband and Delsys.

	Wristband	Delsys ^a^
SNR	**66.96 dB**	22.38 dB
Sampling	1000 Hz	2000 Hz
Channels	8	16
Bandwidth	0–500 Hz	0–500 Hz

^a^ “Delsys” refers to the Delsys electromyography (EMG) system (Delsys Inc., Natick, MA, USA). Bold indicates a better result.

**Table 3:** The results of simulated online testing.

Subject	Day 1	Day 2
1	100.00%	96.00%
2	92.00%	72.00%
3	100.00%	92.00%
4	92.00%	96.00%
5	100.00%	88.00%
6	100.00%	72.00%
7	100.00%	100.00%
8	96.00%	92.00%
9	100.00%	80.00%
10	88.00%	88.00%
Mean	96.80%	87.60%

## Data Availability

Data are not publicly available due to privacy restrictions.

## Abbreviations

The following abbreviations are used in this manuscript:

sEMG	Surface electromyography
SNR	Signal-to-noise ratio
IMU	Inertial measurement unit
CNN	Convolutional neural network
SVM	Support vector machine
FPC	Flexible printed circuit
PCB	Printed circuit board
DRL	Drive electrode
AFE	Analog front-end
MCU	Microcontroller unit
BLE	Bluetooth low energy
GUI	Graphical user interface
